# Funding for malaria control 2006–2010: A comprehensive global assessment

**DOI:** 10.1186/1475-2875-11-246

**Published:** 2012-07-28

**Authors:** David M Pigott, Rifat Atun, Catherine L Moyes, Simon I Hay, Peter W Gething

**Affiliations:** 1 Department of Zoology, Spatial Ecology and Epidemiology Group, University of Oxford, South Parks Road, Oxford, UK; 2 Health Management Group, Imperial College Business School, Imperial College London, London, UK

**Keywords:** Malaria, Equity, Funding, International aid, Policy, Population at risk, *Plasmodium falciparum*, *Plasmodium vivax*

## Abstract

**Background:**

The last decade has seen a dramatic increase in international and domestic funding for malaria control, coupled with important declines in malaria incidence and mortality in some regions of the world. As the ongoing climate of financial uncertainty places strains on investment in global health, there is an increasing need to audit the origin, recipients and geographical distribution of funding for malaria control relative to populations at risk of the disease.

**Methods:**

A comprehensive review of malaria control funding from international donors, bilateral sources and national governments was undertaken to reconstruct total funding by country for each year 2006 to 2010. Regions at risk from *Plasmodium falciparum* and/or *Plasmodium vivax* transmission were identified using global risk maps for 2010 and funding was assessed relative to populations at risk. Those nations with unequal funding relative to a regional average were identified and potential explanations highlighted, such as differences in national policies, government inaction or donor neglect.

**Results:**

US$8.9 billion was disbursed for malaria control and elimination programmes over the study period. Africa had the largest levels of funding per capita-at-risk, with most nations supported primarily by international aid. Countries of the Americas, in contrast, were supported typically through national government funding. Disbursements and government funding in Asia were far lower with a large variation in funding patterns. Nations with relatively high and low levels of funding are discussed.

**Conclusions:**

Global funding for malaria control is substantially less than required. Inequity in funding is pronounced in some regions particularly when considering the distinct goals of malaria control and malaria elimination. Efforts to sustain and increase international investment in malaria control should be informed by evidence-based assessment of funding equity.

## Background

Significant progress has been made towards achieving the Millennium Development Goals [1,2], especially the target for malaria set out in Goal 6.3 of halting and reversing the incidence of malaria by 2015 [3-6]. Success has also been attained by national malaria elimination programmes, with countries such as the United Arab Emirates [7], Morocco [8] and Turkmenistan [9] certified as malaria free between 2007 and 2012. These achievements have been driven by huge increases in the availability of funding for malaria control, and the last decade has seen the increasing prominence of international donors in assisting national governments in control strategies [10,11].

However, international donor support is at a critical juncture. The ongoing global financial crisis and austerity programmes promoted by many governments has meant the trend of increasing international funding has shown signs of significant slowdown [12]. Commitments on future funding are becoming increasingly difficult to secure, with the Global Fund to Fight AIDS, Tuberculosis and Malaria (Global Fund) forced to suspend new projects until 2014 with US$500 million less funding provided in 2011 than that invested in 2010 [13,14]. Faltering funding will, inevitably, jeopardize the progress of recent years in malaria control and raise the likelihood of deteriorating morbidity and mortality trends and the threat of resurgence in transmission [15-17]. In an austere era, sustaining momentum in malaria control requires that limited financial resources are deployed with maximum efficiency, a sentiment emphasized in the most recent Global Fund “Investing for Impact” strategy [18,19]. Moving beyond slogans to achieve this objective requires a comprehensive triangulation of (i) existing funding on malaria control from all sources in each endemic country, (ii) the relative contribution of different national and international funders and (iii) the level of funding relative to populations at risk.

Previous studies have assessed equity in malaria financing on a global scale with a focus on investments by international donors [20,21]. Whilst informative, inferences on overall levels of equity are limited by the omission of data on national government funding. Apparent shortfalls in international assistance may be explained by investment within wealthier nations with adequate internal budgets of their own to support control programmes. Conversely, apparently generous international support to a country may still be inadequate when national governmental contributions to disease control are low. Here, explicit measures of both internal and external funding are incorporated to provide, for the first time, a comprehensive audit of combined malaria control financing. Levels of funding are compared to revised population at risk figures, calculated using the latest 2010 transmission limits for both *Plasmodium falciparum*[22] and *Plasmodium vivax*[23] produced by the Malaria Atlas Project (MAP) [24,25]. This analysis has allowed us to compare levels of governmental support to national control programmes as well as identify those apparently neglected by international donor funding.

## Methods

A review of malaria funding from international donors, non-governmental organisations (NGOs) and government budgets received by each malaria endemic country was undertaken (Additional files 1 and 2). This funding was standardized per capita-at-risk, using combined *P. falciparum* and *P. vivax* transmission limits. National funding was compared to a regional equity level. Each of these components is now described in more detail.

### External funding disbursements

This study attempts to capture as broad a range of funding sources as possible in order to best enumerate the total monies available from external sources for countries to invest in their malaria programmes. Previous studies assessed donor assistance *via* commitments, whereas we have evaluated disbursements wherever possible. The latter presents a more accurate representation of funding within the sector. In several cases, committed funds have been withdrawn before being completely disbursed (e.g. for political, logistical or other reasons relating to improper use of funds or ineffective programmes) or disbursements have occurred over a time period longer than initially anticipated [26]. Disbursements, therefore, allow for a more informed judgement of funding equity to be made, since they best cover the real monies received by each country. It is assumed that all monies disbursed to a country are subsequently spent on national malaria control and elimination programmes.

Detailed information on funding by country was obtained from the following sources: The President’s Malaria Initiative (PMI) up to and including the fiscal year (FY) 2010; the Global Fund up to and including Round 10 grants (decided before December 2010); the Development Assistance Committee (DAC), whose members provide independent bilateral agreements, with data listed from 2005 to 2010; UNICEF (2005–2010); and the Booster Program for Malaria Control in Africa (headed by the World Bank) including Phase 1 funding schemes. The disbursed amounts from the DAC and UNICEF were obtained from the Creditor Reporting System (CRS) [27] using the search criteria “12262: Malaria Control” as the Sector code, and “Disbursements gross (current USD millions)” in the Amount field. For PMI, funding was based upon the Fifth Annual Report (for up to FY 2010) [28] and the Malaria Operational Plans for 2011 [29], whilst Global Fund disbursement data were obtained from the organisation’s country portfolios [30]. World Bank data were collected from the Booster Program grants listing [31], and were assumed to be disbursed evenly across the entire grant period. All currency rates were consistent to the year of disbursement since this allowed for a better understanding of the actual purchasing power these monies provided at that time.

Only those grants that were specifically malaria focused or had clearly defined malaria budgets within an overall larger grant were selected. Although substantial sums are provided for general health-system and infrastructure strengthening and research into malaria, these cannot be directly apportioned to malaria control. Many multilateral grants were therefore excluded, such as those from the Global Alliance for Vaccines and Immunisation (GAVI), the World Bank, the Bill and Melinda Gates Foundation and grants (including many research grants) where no clear evidence of which countries were to benefit. As a result, the data here represent a lower bound estimate for external support levels.

Four grants included in this study were shared between two or more countries described in Additional file 3. In order to gain national subtotals, these regional schemes were divided using final and proposed budgets. Disbursement ratios were assumed constant throughout the grant period.

### Government funding

Grant proposals to the Global Fund were collated [30], using the most up to date information from 83 countries to identify domestic budgets for government funding. This approach was used because these proposals represent the most accessible yet detailed account of national malaria budgets, and hence allowed us to avoid double-counting of funding. It is assumed that such reports represent a valid, accurate and unbiased assessment of government budgets. For the 20 (21%) countries where this was not possible (because no Global Fund support had been requested) we used the World Malaria Report 2011 [32] as the next most reliable reference. If data were missing for a specific year, we used the figures from the adjacent years to make a reasoned estimate by extrapolation. National funding on malaria therefore can be represented by the sum of government budgets and disbursed monies.

### Gross domestic product

Gross Domestic Product (GDP) estimates for 2010 were taken from the World Bank online data resource [33] and divided by the national population, taken from the UN population division. In cases where no 2010 data were present, the 2009 figure was used, or if this was absent, the CIA factbook [34].

### Assessment of populations at risk

Using methods outlined elsewhere, absolute figures for populations at risk of stable and unstable *P. falciparum*[22] and *P. vivax*[23] transmission for 2010 were calculated. Combined *P. falciparum*/*P. vivax* risk for a given population was defined by the highest risk level present for either *P. falciparum* or *P. vivax* (Table 1). Whilst risk for *P. falciparum* affects the entire population, *P. vivax* impacts only Duffy-positive individuals [35,36]. Therefore, in locations where *P. vivax* was stable and *P. falciparum* unstable (marked with * in Table 1) a global map of predicted Duffy negativity prevalence [37] was used to calculate the Duffy-negative population fraction at that location. This fraction was classified as being at unstable risk and the remainder as being at stable risk.

**Table 1:** **Criteria for evaluating population sizes at combined*PfPv*Risk**

	**Stable*Pv*Risk**	**Unstable*Pv*Risk**	**No*Pv*Risk**
**Stable*Pf*Risk**	All Stable Risk	All Stable Risk	All Stable Risk
**Unstable*Pf*Risk**	Duffy Dependent*	All Unstable Risk	All Unstable Risk
**No*Pf*Risk**	Duffy Positives Stable Risk	Duffy Positives Unstable Risk	None

### Assessment of equity

In order to assess the balance of malaria control funding globally, or funding equity, it is necessary to compare each nation to a fixed standard, and analyze what causes variation around this value. Therefore, the globe was divided into three malarious regions, Africa+, Central and South East Asia (CSE Asia) and the Americas (see Additional file 1: for country listings) based upon dominant vector species and other shared epidemiological characteristics [22,38]. Within each region, regional funds per capita-at-risk for the five year period 2006–10 were evaluated by dividing the total regional funding over the five year period by the total population at risk in the area. This average was defined as a theoretical ‘line of equity’ representing the amount that would hypothetically have been disbursed to each country under the assumption of perfect per capita-at-risk equity for each nation. Total disbursements and government funding within each country over the five-year study period were compared to this equity line in order to assess equity on the national scale. The per capita-at-risk metric simplifies the distinct needs of each transmission group identified, yet insufficient data on what necessary funding is required by each category prevents a more detailed analysis.

## Results

### Summary of global funding 2006–2010

**Figure 1:**
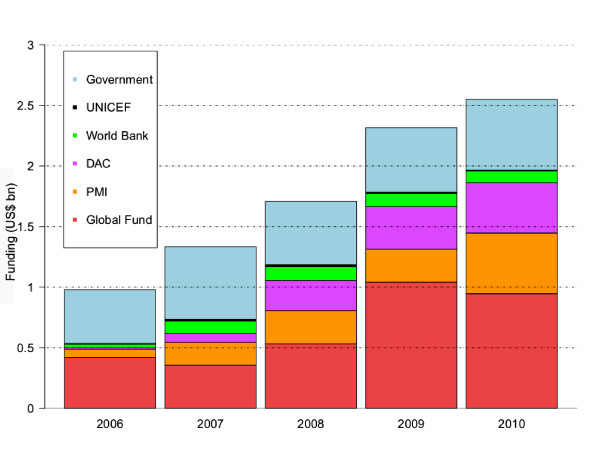
**Bar chart of amounts disbursed by different funding sources 2006–2010.** The blue “Government” portion refers to the amounts disbursed by national governments within their own country.

Total funding for malaria control increased year-on-year throughout the 2006–2010 period, from US$980 million in 2006 to US$2.55 billion in 2010 (Figure 1). Funding from PMI has risen from US$65 million in its first year of full funding (2006) to US$500 million across 17 countries in 2010, with similar figures for 2011 and proposed for the FY2012 [39]. Likewise, the annual disbursement from the Global Fund has increased dramatically from its inception, where the Round 1 commitments in 2002 for malaria were US$68 million, to releasing over US$1 billion in 2009 and close to that amount again in 2010. This trend is further seen in the amount donated by DAC nations, which displayed year-on-year increases throughout the study period, with just over US$400 million donated in 2010. UNICEF malaria funding has been in decline since its peak from 2007 and is now just around US$8 million, representing one of the smaller funding streams investigated in this study. There has been a decline in annual disbursements from the World Bank’s Booster Scheme with most national grants now being complete, or in their closing stages. When considered together, external funding has nearly quadrupled from US$535 million in 2006, to just under US$2 billion in 2010. Over the same period, the total domestic government funding across the globe has remained relatively consistent, between US$500-600 million, but represents a declining proportion of total global funding.

Some interesting findings in national-level funding exist. Over the five-year period studied, eight malaria endemic countries had received no international support (Table 2) and four others received negligible amounts from the international community (under $50,000 cumulative) (Table 2). These countries are all outside Africa and are characterized by small populations at risk, a predominance of *P. vivax* and above-average GDP per capita.

**Table 2:** Countries where government funding represents the major funding stream


Countries receiving no external support	Belize, Costa Rica, Iraq, Malaysia, Panama, Paraguay, Republic of Korea, Saudi Arabia, Turkey
Countries receiving < $50000 over 2006-10	Argentina, Cape Verde, El Salvador, Mexico
Countries >50% of total funding from government sources, 2006-10	Argentina, Belize, Bhutan, Botswana, Brazil, Cape Verde, Colombia, Costa Rica, Ecuador, El Salvador, Guatemala, Guyana, India, Iran, Iraq, Malaysia, Mexico, Namibia, Nicaragua, Pakistan, Panama, Paraguay, Peru, Republic of Korea, Saudi Arabia, South Africa, Sri Lanka, Thailand, Togo, Turkey, Uzbekistan, Venezuela, Vietnam

**Figure 2:**
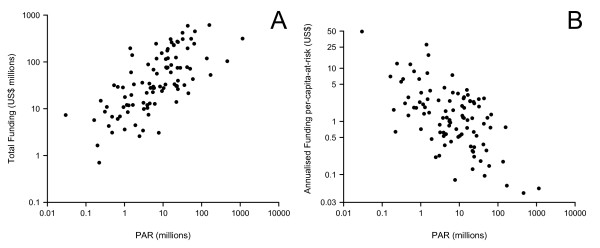
**Scatter plots of populations at risk and patterns of funding.** Panel A shows total funding over the period 2006–2010 against size of the population at risk of *PfPv* per country. Panel B shows annualized funding per capita against size of the population at risk of *PfPv* per country.

Globally, there is an approximately linear relationship between the national-level size of the population at risk and total national-level funding (Figure 2A). Whilst there is broad correspondence between the size of population at risk in each country and the total amount spent, the financing does not scale proportionately: funding per capita-at-risk decreases as the total population at risk increases, ranging from US$49 per capita-at-risk in Suriname to as low as US$0.04 in China (Figure 2B). This echoes data shown in the World Malaria Report 2009, therefore suggesting that in spite of the additional funding over the period 2007–2010, little has changed in this regard [40].

As might be expected, comparison by country of the relative contributions of government versus international support revealed large differences in the degree of self-reliance. Whilst most countries in which governmental funding exceeded international support were those with higher GDP per capita (Table 2), there were some notable exceptions that are discussed in more detail below.

Patterns of DAC disbursements remain strongly linked to colonial legacies (Additional file 4). Portugal donated 100% of its contributions to former colonies, and over 90% of British disbursements were to Commonwealth and former colonies. Over 80% of both Belgian and French disbursements were similarly directed towards their former territories. Australia shows an equivalent bias with respect to its regional neighbours, with 99% of support being directed towards the Asia-Pacific Region. Interestingly, the additional international support of the USA was provided mainly in support of those already receiving PMI grants, predominantly in Sub-Saharan Africa, strengthening their existing activities.

Table 3 summarizes the distribution of funding by global region. Over 70% of all finance and 87% of all external support for malaria control was spent in Africa. Asia and the Americas received 17.93% and 8.74% of funding respectively. These patterns are in contrast to global distributions of populations at risk. In absolute numbers, CSE Asia accounts for 70% of global population at any risk of malaria and 55% at stable risk. These differences are further highlighted when comparing the regional levels of equity. The Africa+ region averages US$1.60 per person-at-risk per year, compared to US$0.93 in the Americas and US$0.13 in CSE Asia. Even with the exclusion of China and India, the per capita average in CSE Asia only increases to US$0.33.

**Table 3:** Regional proportions of funding and populations at risk

**Region**	**Total funding (2006–10)**	**External funding (2006–2010)**	**Population at risk (Total)**	**Population at risk (Stable)**
Africa+	73.33%	86.89%	24.53%	41.43%
CSE Asia	17.93%	11.47%	70.40%	55.27%
Americas	8.74%	1.64%	5.07%	3.30%

#### The Americas

**Figure 3:**
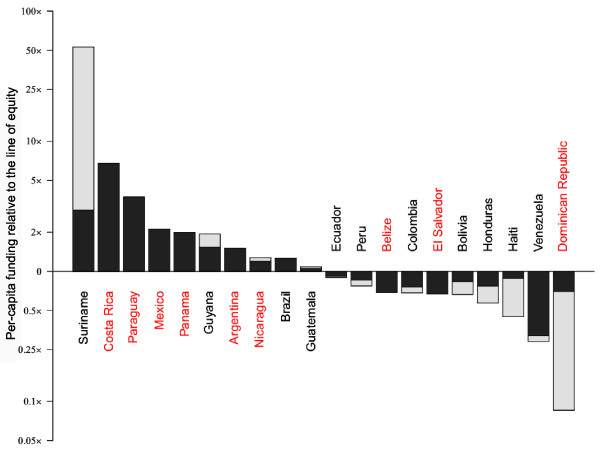
**Bar chart of assessment of equitable funding in the Americas.** Bars represent annualized per capita funding in each malaria endemic country in the region relative to the line of equity, defined as the theoretical level if regional funding was perfectly distributed to countries according to populations at risk. The divisions in the bar demonstrate the relative external and government contribution. For the Americas, the line of equity is US$0.93 per capita per year. Countries labelled in red are in the process of moving from controlled low-endemic malaria to elimination [53]. The y axis indicates by what factor the national per capita-at-risk funding differs from the line of equity.

Funding for malaria in the Americas region is dominated by government funding (Figure 3). Of the 20 malaria endemic countries in the region, seven received less than US$50,000 of international funding over the study period. External support, when it has been supplied, is predominately by the Global Fund and for targeted use in remote, high-risk communities of the Amazon Basin, such as in Suriname, Guyana, Brazil and the Multi-country Americas (Andean) Group (defined in Additional file 3). Where these communities represent a significant proportion of the total population at risk, above equitable levels of funding result; where not, the government is expected to extend such support across the rest of the country. Whilst Brazil, with the strongest economy in the region, is capable of such an undertaking, Colombia, Ecuador and Peru have not been able to do so, as shown by their position below the line of equity for the Americas region in Figure 3.

For several countries, the high levels of per capita-at-risk funding correspond to the transition from low-endemic control to elimination programmes. Costa Rica, Paraguay, Mexico, Panama and Argentina all have relatively small populations at risk, experience mainly unstable transmission risk and their national governments have supported an elimination agenda, resulting in greater than “equitable” funding.

Many of the countries that are struggling to maintain per capita-at-risk funding parity are those that are currently more focused on controlling malaria rather than elimination. Whilst in some cases it could be argued that national government funding is lower than expected (for instance compare Bolivian and Honduran government funding with that of Nicaragua – Additional file 1), this is not the case for all, such as with Haiti and the Dominican Republic. In the latter examples, we note that grants were in place for these countries in 2011, which will help to counter the apparent inequity. Haiti, for instance, is set to receive US$18 million, greater than its cumulative total of the previous five years.

#### Africa+

**Figure 4:**
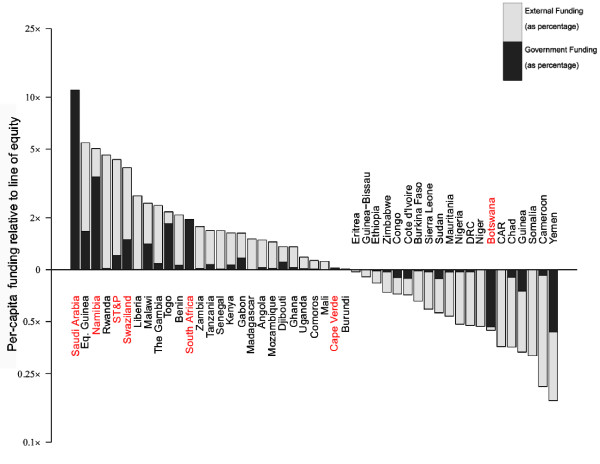
**Bar chart of assessment of equitable funding in Africa+.** Details as described for Figure 3. For Africa+, the line of equity is US$1.60.

In Africa+, funding is dominated by international support (Figure 4). The majority of countries in the Africa + region are control-focused, with strategies heavily funded by external donors. It is therefore unsurprising to see patterns of inequity driven by patterns in donor assistance. Whilst the Global Fund is geographically comprehensive in its funding strategy, providing funding largely in proportion to populations at risk, the disbursement patterns of the DAC and PMI are more targeted and thus introduce inequity in the continent-wide pattern of funding. Of the 17 countries that received PMI support by 2010, 14 have higher than equitable funding on malaria when compared to their African neighbours (Additional files 1 and 2). Similar trends are apparent when DAC recipients are considered. The net result is that, for countries outside of these funding streams (for example, Cameroon, that relies primarily on the Global Fund), funding per capita-at-risk is below the regional average.

Some countries with proportionally large government funding stand out, in terms of equitable levels of funding, size of population at risk or levels of risk compared to the rest of the region. Namibia and South Africa have supported their goals of elimination primarily through domestic funding. Saudi Arabia similarly has higher than equitable funding, consistent with the country’s relative wealth and small population at risk, and is thus well placed to target elimination through largely domestically financed programmes. Swaziland and São Tomé and Príncipe, in contrast, are supported in their elimination efforts predominantly by the Global Fund and, given their small populations at risk, have higher than equitable funding. In contrast, Botswana receives little international support and is ineligible for the Global Fund ‘general pool’ (due to its classification as an Upper-Middle Income country) meaning it cannot achieve parity with the rest of the region despite above-average government investment.

#### Central and South East Asia

**Figure 5:**
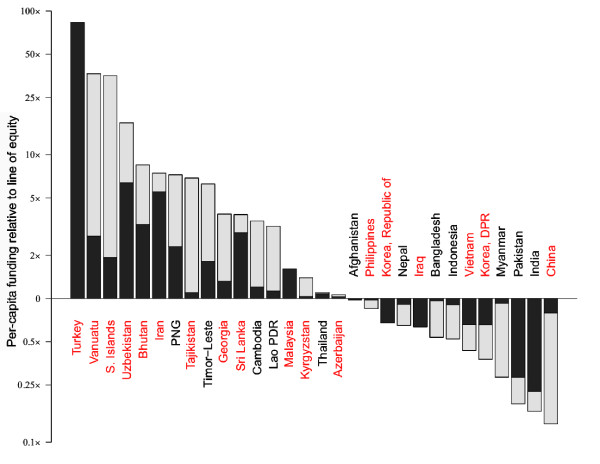
**Bar chart of assessment of equitable funding in CSE Asia.** Details as described for Figure 3. For CSE Asia, the line of equity is US$0.33.

The CSE Asia region is characterized by very diverse levels of funding (Figure 5). There are strong ties between the ranking and the size of the population at risk, echoing the trend of Figure 2B. Of the countries with above-average levels of funding in the region, over two thirds (12 of 17) are pursuing elimination. This is achieved through either strong governmental commitment, as in Turkey and Iran, or *via* concerted elimination programmes backed by the international community, as with Vanuatu, the Solomon Islands and the former Soviet states. That said, there are still some nations which have commenced elimination programmes but are funding below equitable levels, such as the Philippines or Vietnam. Levels of funding above the equity line were found in those countries associated with the emergence of artemisinin resistance, particularly Cambodia [41]. Investment in resistance containment has been led by the international community *via* targeted Global Fund grants, and by the Thai government. Recent evidence of resistance emergence in Myanmar [42] is worrying however, particularly given the low levels of funding there. The Global Fund has been a lone significant international donor in the region but, as of 2011, the PMI has become operational in the Greater Mekong sub-region, with potentially important consequences for the containment of artemisinin resistance [41].

Countries in the region with a higher per capita GDP tended to have greater government funding relative to donor assistance with two important exceptions: Indonesia and China. Both countries receive substantial funding from the Global Fund, but provide comparatively small government funding in support, meaning the funding per capita-at-risk is surprisingly low. In contrast, a high proportion of the funding in India and Pakistan comes from internal resources relative to international aid, yet these countries still only achieve minimal per capita funding due to their significant population sizes.

## Discussion

This analysis indicates that external funding for malaria was approximately US$2 billion in 2010 which, when combined with national government funding, led to a total of some US$2.5 billion spent that year on countering malaria globally. However, assessments have placed annual funding needs for malaria control at between four and six billion dollars [17,43]: a stark deficit persists, therefore, between what is needed and what is provided.

The distinction between equity and adequacy is critically important. It seems clear that few countries receive adequate levels of funding but, even in the context of this global inadequacy, there are marked discrepancies when funding is compared to populations at risk. Combatting malaria must ultimately be considered as a global endeavour and the challenge to the international community is to raise overall funding levels substantially whilst seeking to address existing inequities. The inherently interconnected nature of malaria epidemiology is most clearly exemplified in elimination settings: the threat of importation or potential for vector dispersal from a neighbouring country will always represent a risk [44]. Hence, ongoing adequate and equitable funding is needed if the gains are to be sustained and progress to date not undermined.

This analysis backs up the view that the trend for increasing funding in the malaria sector is levelling off, suggesting this deficit will remain [17]. In this context, the onus lies on funders to maintain existing levels of funding and recognize that these levels must be increased if international targets in burden reduction and elimination are to be realized. Crucially, however, it can be seen that governments of malaria endemic countries across the GDP spectrum have committed smaller proportions of their own national budgets than might be expected when compared to regional neighbours. Individual country-level assessments must be performed to determine which sector is most culpable for any deficit in the context of regional patterns of funding. In some cases, increases in both internal and external funding are required. There is no evidence to suggest a significant displacement of government funding by international support [45]; indeed the global level of government funding remained relatively consistent between 2006 and 2010 despite large increases in donor assistance.

The observed inverse relationship between population at risk and per capita-at-risk funding also presents a concern (Figure 2B) [40]. This relationship implies that funding to those countries with the largest number of people at risk is not scaled sufficiently. Whilst, in theory, economies of scale may mean that protection of large populations becomes less costly per capita, there is little evidence [46] to suggest this would compensate for the funding disparities we observe. The marked decline observed in per capita funding thus likely represents a systematic under-funding in those countries with the largest populations at risk.

The funding of disease control to minimize morbidity and mortality burdens rightly dominates global funding patterns. Africa, with the most intense transmission [22], largest burden of mortality [3] and many of the world’s poorest nations [33], receives close to 70% of all disbursements. The region has also seen some of the greatest successes against malaria, as well as some of the most notable setbacks when funding has been withdrawn [15,16]. In contrast, the CSE Asia region represents a significant proportion of the global at-risk population, but displays highly variable per capita-at-risk support that is markedly inequitable in some cases. An important aspect of these disparities is the role of *P. vivax* malaria which arguably receives disproportionately little donor funding for control, especially given a mounting body of evidence suggesting its clinical importance has been underestimated substantially [47-52]. When comparing such regions it must be kept in mind that the per capita-at-risk metric does not take into account the varying epidemiology that these regions represent. Doing so requires a far greater assessment of the effectiveness of different strategies in different scenarios, beyond the scope of this study. Future investigations of this kind will better enable future comparative assessments of equity across the globe.

Of the countries targeting malaria elimination, over two thirds (24 out of 35) have above-equitable levels of funding. Due to small populations at risk, consideration of absolute monies spent, as well as per capita amounts in these cases is needed. Of the 35 nations identified as pursuing malaria elimination [53], only 15 have an annual per capita funding greater than US$2. This result, combined with the total levels of funding in these countries, compares poorly to studies that have investigated the necessary amounts of investment required for elimination [54]. Many of those nations that are funding at an appropriate level to make substantial progress towards malaria elimination are either financing the operation themselves (such as Turkey or Saudi Arabia), or are part of a concerted international elimination effort (such as Solomon Islands and Vanuatu).

An interesting finding is that patterns of DAC disbursements remain strongly linked to colonial legacies, especially for Great Britain, Belgium, France and Portugal – countries which are signatories to the Paris Declaration and Accra Agenda for Action on Aid Effectiveness that foster untied aid [55]. Similarly, disbursements from USAID are primarily directed to reinforcing PMI investments. In attempting to achieve adequacy of funding on malaria control in specific countries, these have inadvertently become drivers of uneven funding on a regional and global level.

## Conclusions

Global funding levels for malaria are in an increasingly precarious state, and we must consolidate the gains that have been facilitated by this financing over the last decade. To do so, commitments to existing programmes that have proven successful must be reaffirmed, as well as assessing whether existing patterns of funding are the most appropriate. This analysis shows that, globally, inadequate levels of funding persist, and that there are large inequalities, which vary in importance and ease of resolution, whether through increased government support or international assistance. In some cases, international funding remains tied to colonial legacies rather than disease burden. It is crucial that such imbalances are addressed in attempting to secure adequate global funding for malaria. The very large populations at risk in CSE Asia suggest that this region should become more prominent in discussions on future investment, but increased funding should not be achieved through the diversion of funds from other schemes. “Investing for impact” represents a new principle for any future funding. Targeting the most at-risk populations and tailoring disbursement of funds to a country’s needs, whilst still framing funding decisions within the context of global disease distribution, is key. Continued monitoring and spatial assessments of national financial and disease status will enable this and allow us to maximize the effectiveness of future funding.

## Abbreviations

CSE Asia: Central and South East Asia; DAC: Development Assistance Committee; GDP: Gross Domestic Product; Global Fund: The Global Fund to Fight AIDS, Tuberculosis and Malaria; MAP: Malaria Atlas Project; *PfPv*: Combined *P*. *falciparum* and *P*. *vivax*; PMI: President’s Malaria Initiative; UNICEF: United Nations Children’s Fund.

## Competing interests

RA is a former Director of the Strategy, Performance and Evaluation cluster at the Global Fund.

## Authors’ contributions

RA, SIH and PWG conceived the analysis. DMP and PWG collated necessary data and led the analysis. DMP and PWG wrote the first draft of the manuscript. All authors contributed to refining the analysis and the final version of the manuscript.

## Supplementary Material

Additional file 1 Funding for malaria by source. Click here for file

Additional file 2 National Funding and Populations-at-Risk. Click here for file

Additional file 3 Multi-Country Grants and Their Divisions. Click here for file

Additional file 4 Funding patterns in selected DAC donors. Click here for file
