# Supplementary material for: Funding for malaria control 2006–2010: A comprehensive global assessment
Source: Malar J. 2012 Jul 28;11:246. doi: 10.1186/1475-2875-11-246 (PMC3444429; doi:10.1186/1475-2875-11-246)
Supplement: Additional file 1 — Funding for malaria by source. [file 1475-2875-11-246-S1.docx]

| **Additional file 1**Funding for malaria by source (2006-2010) | | | | | | | | | | | | | | | | | |  | |
| --- | --- | --- | --- | --- | --- | --- | --- | --- | --- | --- | --- | --- | --- | --- | --- | --- | --- | --- | --- |
| (all US$) | | **GDP per capita** | | | **Government** | | ***Global Fund^[1]^*** | | ***PMI^[2]^*** | | | ***DAC^[3]^*** | ***UNICEF^[4]^*** | | ***World Bank*** | | **External Funds** | | |
| **Americas region** | |  | | |  | |  | |  | | |  |  | |  | |  | | |
| Argentina | | 9,123.74 | | | 8,424,415 | | 0 | | 0 | | | 33,175 | 0 | | 0 | | 33,175 | | |
| Belize | | 4,593.63 | | | 704,059 | | 0 | | 0 | | | 0 | 0 | | 0 | | 0 | | |
| Bolivia | | 1,992.59 | | | 5,363,463 | | 6,631,272 | | 0 | | | 1,433 | 0 | | 0 | | 6,632,705 | | |
| Brazil | | 10,710.07 | | | 301,181,100 | | 10,361,470 | | 0 | | | 244,865 | 0 | | 0 | | 10,606,335 | | |
| Colombia | | 6,225.08 | | | 54,531,825 | | 20,288,108 | | 0 | | | 0 | 0 | | 0 | | 20,288,108 | | |
| Costa Rica | | 7,419.02 | | | 28,495,000 | | 0 | | 0 | | | 0 | 0 | | 0 | | 0 | | |
| Dominican Republic | | 5,195.45 | | | 444,351 | | 2,603,831 | | 0 | | | 13,453 | 0 | | 0 | | 2,617,284 | | |
| Ecuador | | 4,072.66 | | | 18,033,750 | | 4,550,798 | | 0 | | | 0 | 0 | | 0 | | 4,550,798 | | |
| El Salvador | | 3,519.42 | | | 9,864,000 | | 0 | | 0 | | | 0 | 1,440 | | 0 | | 1,440 | | |
| Guatemala | | 2,862.62 | | | 17,367,015 | | 10,160,726 | | 0 | | | 174,858 | 0 | | 0 | | 10,335,584 | | |
| Guyana | | 2,945.16 | | | 4,455,000 | | 2,390,397 | | 0 | | | 0 | 0 | | 0 | | 2,390,397 | | |
| Haiti | | 671.43 | | | 1,945,000 | | 10,721,655 | | 0 | | | 0 | 27,860 | | 0 | | 10,749,515 | | |
| Honduras | | 2,026.22 | | | 4,800,579 | | 5,589,508 | | 0 | | | 0 | 0 | | 0 | | 5,589,508 | | |
| Mexico | | 9,166.23 | | | 114,948,775 | | 0 | | 0 | | | 0 | 46,600 | | 0 | | 46,600 | | |
| Nicaragua | | 1,131.82 | | | 20,955,000 | | 6,906,715 | | 0 | | | 0 | 0 | | 0 | | 6,906,715 | | |
| Panama | | 7,614.01 | | | 11,817,743 | | 0 | | 0 | | | 0 | 0 | | 0 | | 0 | | |
| Paraguay | | 2,862.28 | | | 17,740,426 | | 0 | | 0 | | | 0 | 0 | | 0 | | 0 | | |
| Peru | | 5,291.04 | | | 18,000,000 | | 6,782,441 | | 0 | | | 5,033,421 | 6,580 | | 0 | | 11,822,442 | | |
| Suriname | | 6,197.38 | | | 2,000,000 | | 5,332,757 | | 0 | | | 0 | 0 | | 0 | | 5,332,757 | | |
| Venezuela | | 13,383.49 | | | 28,127,386 | | 2,466,342 | | 0 | | | 21,129 | 0 | | 0 | | 2,487,471 | | |
| **CSE Asia region** | |  | | |  | |  | |  | | |  |  | |  | |  | | |
| Afghanistan | | 548.94 | | | 2,250,000 | | 37,535,424 | | 0 | | | 0 | 0 | | 0 | | 37,535,424 | | |
| Azerbaijan | | 5,560.88 | | | 3,310,000 | | 4,128,827 | | 0 | | | 0 | 0 | | 0 | | 4,128,827 | | |
| Bangladesh | | 673.04 | | | 1,830,000 | | 30,030,047 | | 0 | | | 102,112 | 0 | | 0 | | 30,132,159 | | |
| Bhutan | | 2,088.43 | | | 3,739,000 | | 3,009,604 | | 0 | | | 0 | 0 | | 0 | | 3,009,604 | | |
| Cambodia | | 802.32 | | | 11,134,006 | | 64,870,006 | | 0 | | | 6,725 | 180 | | 0 | | 64,876,911 | | |
| China | | 4,382.67 | | | 11,904,945 | | 91,749,492 | | 0 | | | 0 | 900 | | 0 | | 91,750,392 | | |
| Georgia | | 2,680.77 | | | 624,138 | | 2,461,891 | | 0 | | | 0 | 0 | | 0 | | 2,461,891 | | |
| India | | 1,411.88 | | | 259,799,844 | | 55,368,082 | | 0 | | | 80,265 | 97,570 | | 0 | | 55,545,917 | | |
| Indonesia | | 2,945.58 | | | 18,555,556 | | 99,127,214 | | 0 | | | 1,030,754 | 88,440 | | 0 | | 100,246,408 | | |
| Iran | | 4,474.77 | | | 30,606,375 | | 5,398,910 | | 0 | | | 4,327 | 0 | | 0 | | 5,403,237 | | |
| Iraq | | 2,593.82 | | | 2,587,474 | | 0 | | 0 | | | 0 | 0 | | 0 | | 0 | | |
| Korea, Democratic People's Republic of | | 1,158.29 | | | 6,000,000 | | 7,933,014 | | 0 | | | 0 | 0 | | 0 | | 7,933,014 | | |
| Korea, Republic of | | 21,054.54 | | | 3,419,891 | | 0 | | 0 | | | 0 | 0 | | 0 | | 0 | | |
| Kyrgyzstan | | 865.38 | | | 399,100 | | 4,044,819 | | 0 | | | 0 | 0 | | 0 | | 4,044,819 | | |
| Lao People's Democratic Republic | | 1,208.26 | | | 3,001,651 | | 26,638,391 | | 0 | | | 9,618 | 0 | | 0 | | 26,648,009 | | |
| Malaysia | | 8,373.08 | | | 74,791,841 | | 0 | | 0 | | | 0 | 0 | | 0 | | 0 | | |
| Myanmar | | 1,046.64 | | | 1,300,000 | | 12,980,805 | | 0 | | | 3,502,463 | 3,639,580 | | 0 | | 20,122,848 | | |
| Nepal | | 524.08 | | | 5,780,602 | | 20,433,609 | | 0 | | | 0 | 0 | | 0 | | 20,433,609 | | |
| Pakistan | | 1,006.95 | | | 39,413,000 | | 13,118,357 | | 0 | | | 0 | 114,930 | | 0 | | 13,233,287 | | |
| Papua New Guinea | | 1,382.28 | | | 28,672,550 | | 39,467,298 | | 0 | | | 204,035 | 12,210 | | 0 | | 39,683,543 | | |
| Philippines | | 2,140.12 | | | 10,500,000 | | 58,625,897 | | 0 | | | 2,332,532 | 0 | | 0 | | 60,958,429 | | |
| Solomon Islands | | 1,261.04 | | | 5,791,258 | | 13,276,005 | | 0 | | | 12,663,950 | 0 | | 0 | | 25,939,955 | | |
| Sri Lanka | | 2,375.45 | | | 60,000,000 | | 16,629,084 | | 0 | | | 0 | 0 | | 0 | | 16,629,084 | | |
| Tajikistan | | 819.99 | | | 511,315 | | 10,316,079 | | 0 | | | 0 | 0 | | 0 | | 10,316,079 | | |
| Thailand | | 4,612.80 | | | 58,862,505 | | 17,173,189 | | 0 | | | 1,273,432 | 0 | | 0 | | 18,446,621 | | |
| Timor-Leste | | 623.47 | | | 3,903,200 | | 6,631,864 | | 0 | | | 1,492,717 | 0 | | 0 | | 8,124,581 | | |
| Turkey | | 10,106.39 | | | 195,867,260 | | 0 | | 0 | | | 0 | 0 | | 0 | | 0 | | |
| Uzbekistan | | 1,420.37 | | | 5,675,438 | | 2,957,703 | | 0 | | | 0 | 0 | | 0 | | 2,957,703 | | |
| Vanuatu | | 3,041.73 | | | 4,105,868 | | 6,839,155 | | 0 | | | 3,877,665 | 0 | | 0 | | 10,716,820 | | |
| Viet Nam | | 1,178.98 | | | 21,562,500 | | 21,437,011 | | 0 | | | 0 | 0 | | 0 | | 21,437,011 | | |
| **Additional file 1**Funding for malaria by source (2006-2010) | | | | | | | | | |  | | | | | | | | | |
| (all US$) | | **GDP per capita** | | | **Government** | | ***Global Fund^[1]^*** | | ***PMI^[2]^*** | | | ***DAC^[3]^*** | ***UNICEF^[4]^*** | | ***World Bank*** | | **External Funds** | | |
| **Africa + region** | |  | | |  | |  | |  | | |  |  | |  | |  | | |
| Angola | | 4,422.54 | | | 17,500,000 | | 42,494,446 | | 99,046,000 | | | 64,944,738 | 3,112,520 | | 0 | | 209,597,704 | | |
| Benin | | 749.51 | | | 12,833,795 | | 29,217,092 | | 54,061,000 | | | 27,861,040 | 1,733,580 | | 28,416,667 | | 141,289,379 | | |
| Botswana | | 7,402.93 | | | 3,394,827 | | 0 | | 0 | | | 0 | 210,140 | | 0 | | 210,140 | | |
| Burkina Faso | | 535.58 | | | 3,248,858 | | 66,696,217 | | 0 | | | 7,963,225 | 1,428,210 | | 6,576,922 | | 82,664,574 | | |
| Burundi | | 192.12 | | | 3,245,880 | | 36,147,767 | | 0 | | | 13,296,476 | 3,411,260 | | 0 | | 52,855,503 | | |
| Cameroon | | 1,142.59 | | | 1,600,715 | | 31,142,976 | | 0 | | | 410,261 | 216,290 | | 0 | | 31,769,527 | | |
| Cape Verde | | 3,322.77 | | | 1,619,663 | | 0 | | 0 | | | 22,551 | 0 | | 0 | | 22,551 | | |
| Central African Republic | | 457.39 | | | 41,738 | | 11,760,854 | | 0 | | | 0 | 1,171,070 | | 0 | | 12,931,924 | | |
| Chad | | 675.83 | | | 3,212,922 | | 27,717,061 | | 0 | | | 0 | 2,014,912 | | 0 | | 29,731,973 | | |
| Comoros | | 736.44 | | | 362,397 | | 5,641,387 | | 0 | | | 0 | 47,900 | | 0 | | 5,689,287 | | |
| Congo | | 2,942.84 | | | 7,187,341 | | 11,944,189 | | 0 | | | 1,963,992 | 748,780 | | 0 | | 14,656,961 | | |
| Cote d'Ivoire | | 1,154.14 | | | 42,461,477 | | 79,477,156 | | 0 | | | 1,915,364 | 145,990 | | 0 | | 81,538,510 | | |
| Democratic Republic of Congo | | 199.27 | | | 10,000,000 | | 145,112,573 | | 18,000,000 | | | 32,794,887 | 7,175,820 | | 31,607,268 | | 234,690,548 | | |
| Djibouti | | 1,180.42 | | | 1,392,460 | | 2,611,945 | | 0 | | | 0 | 270,240 | | 0 | | 2,882,185 | | |
| Equatorial Guinea | | 19,997.84 | | | 8,878,011 | | 20,406,808 | | 0 | | | 0 | 4,480 | | 0 | | 20,411,288 | | |
| Eritrea | | 402.96 | | | 1,773,963 | | 30,665,177 | | 0 | | | 0 | 235,040 | | 1,766,667 | | 32,666,884 | | |
| Ethiopia | | 358.25 | | | 37,043,484 | | 240,429,581 | | 79,801,000 | | | 55,069,461 | 4,069,500 | | 33,700,000 | | 413,069,542 | | |
| Gabon | | 8,642.80 | | | 6,337,440 | | 13,304,712 | | 0 | | | 70 | 1,650 | | 0 | | 13,306,432 | | |
| Ghana | | 1,283.46 | | | 27,186,000 | | 87,142,123 | | 76,640,000 | | | 66,002,836 | 2,593,550 | | 6,086,955 | | 238,465,464 | | |
| Guinea | | 451.89 | | | 7,118,224 | | 13,177,292 | | 0 | | | 1,307,301 | 1,432,893 | | 4,571,706 | | 20,489,192 | | |
| Guinea-Bissau | | 579.79 | | | 500,000 | | 10,960,575 | | 0 | | | 0 | 573,900 | | 0 | | 11,534,475 | | |
| Kenya | | 775.28 | | | 54,113,710 | | 141,619,362 | | 91,058,000 | | | 113,819,474 | 442,660 | | 18,452,381 | | 365,391,877 | | |
| Liberia | | 246.91 | | | 319,591 | | 23,667,099 | | 44,699,000 | | | 19,236,382 | 451,300 | | 0 | | 88,053,781 | | |
| Madagascar | | 421.00 | | | 1,141,391 | | 98,033,869 | | 74,631,000 | | | 51,508,248 | 5,009,250 | | 0 | | 229,182,367 | | |
| Malawi | | 342.68 | | | 118,405,968 | | 42,133,044 | | 83,099,000 | | | 57,936,573 | 254,380 | | 5,000,000 | | 188,422,997 | | |
| Mali | | 601.92 | | | 1,063,347 | | 12,652,150 | | 65,269,000 | | | 33,348,228 | 37,600 | | 7,810,000 | | 119,116,978 | | |
| Mauritania | | 1,051.02 | | | 716,270 | | 4,330,589 | | 0 | | | 0 | 63,248 | | 8,286,218 | | 12,680,055 | | |
| Mozambique | | 409.83 | | | 13,381,918 | | 76,452,265 | | 101,797,000 | | | 79,936,178 | 1,000,180 | | 0 | | 259,185,623 | | |
| Namibia | | 5,330.18 | | | 45,813,455 | | 14,094,700 | | 0 | | | 0 | 2,070 | | 0 | | 14,096,770 | | |
| Niger | | 357.71 | | | 302,800 | | 40,003,305 | | 0 | | | 6,403,198 | 3,940,340 | | 9,230,770 | | 59,577,613 | | |
| Nigeria | | 1,222.48 | | | 25,071,400 | | 282,863,339 | | 18,000,000 | | | 68,691,247 | 1,682,890 | | 217,777,777 | | 589,015,253 | | |
| Rwanda | | 529.71 | | | 2,900,000 | | 116,206,881 | | 72,641,000 | | | 53,069,166 | 155,960 | | 0 | | 242,073,007 | | |
| Sao Tome and Principe | | 1,189.90 | | | 742,945 | | 4,789,700 | | 0 | | | 167,673 | 13,330 | | 0 | | 4,970,703 | | |
| Saudi Arabia | | 15,835.94 | | | 139,699,086 | | 0 | | 0 | | | 0 | 0 | | 0 | | 0 | | |
| Senegal | | 1,041.85 | | | 2,500,000 | | 32,532,473 | | 77,438,000 | | | 49,294,386 | 850,820 | | 11,729,908 | | 171,845,587 | | |
| Sierra Leone | | 324.67 | | | 1,555,496 | | 13,347,091 | | 0 | | | 11,055,525 | 1,758,950 | | 0 | | 26,161,566 | | |
| Somalia | | 254.21 | | | 0 | | 21,781,142 | | 0 | | | 2,053,785 | 29,730 | | 0 | | 23,864,657 | | |
| South Africa | | 7,254.81 | | | 259,064,906 | | 2,687,398 | | 0 | | | 105 | 0 | | 0 | | 2,687,503 | | |
| Sudan | | 1,584.64 | | | 40,185,187 | | 110,703,082 | | 0 | | | 28,705,996 | 813,670 | | 14,750,000 | | 154,972,748 | | |
| Swaziland | | 3,073.44 | | | 3,231,360 | | 7,687,785 | | 0 | | | 0 | 2,110 | | 0 | | 7,689,895 | | |
| Tanzania | | 514.18 | | | 79,719,592 | | 209,987,087 | | 163,225,000 | | | 115,512,893 | 1,214,760 | | 21,875,000 | | 511,814,740 | | |
| The Gambia | | 466.63 | | | 3,087,924 | | 29,890,176 | | 0 | | | 0 | 195,580 | | 0 | | 30,085,756 | | |
| Togo | | 523.14 | | | 93,707,542 | | 23,925,034 | | 0 | | | 20,701 | 192,154 | | 0 | | 24,137,889 | | |
| Uganda | | 508.93 | | | 24,000,000 | | 111,290,939 | | 109,422,000 | | | 59,527,810 | 3,194,060 | | 0 | | 283,434,809 | | |
| Yemen | | 1,096.15 | | | 14,542,012 | | 16,114,008 | | 0 | | | 0 | 0 | | 0 | | 16,114,008 | | |
| Zambia | | 1,237.18 | | | 5,798,000 | | 46,032,137 | | 72,308,000 | | | 45,614,090 | 1,093,230 | | 18,837,210 | | 183,884,667 | | |
| Zimbabwe | | 594.52 | | | 7,900,000 | | 63,989,100 | | 0 | | | 1,801,532 | 392,400 | | 0 | | 66,183,032 | | |
|  |  | | | | | | | | | | | | | | | | | | |
| ^[1]^The Global Fund to Fight AIDS, Tuberculosis and Malaria ^[2]^President’s Malaria Initiative ^[3]^Development Assistance Committee ^[4]^United Nations Children’s Fund | | | | | | | | | | | | | | | | | | | |
|  | | |  |  | |  | |  | | |  | | |  | |  | | |  |
