# Supplementary material for: Funding for malaria control 2006–2010: A comprehensive global assessment
Source: Malar J. 2012 Jul 28;11:246. doi: 10.1186/1475-2875-11-246 (PMC3444429; doi:10.1186/1475-2875-11-246)
Supplement: Additional file 2 — National Funding and Populations-at-Risk. [file 1475-2875-11-246-S2.doc]

| **Additional file 2**National Funding and Populations-at-Risk (2006-2010) | | | | | | | | | |
| --- | --- | --- | --- | --- | --- | --- | --- | --- | --- |
|  | **Total Funds** | **Stable *PfPv*PAR^[1,2]^** | | **Total *PfPv*PAR** | | **Total Per-Capita** | | **Annualised Total Per-Capita** |  |
| **Americas region** |  |  | |  | |  | |  |  |
| Argentina | 8,457,590 | 366,401 | | 1,215,614 | | 6.96 | | 1.39 |  |
| Belize | 704,059 | 220,835 | | 220,865 | | 3.19 | | 0.64 |  |
| Bolivia | 11,996,168 | 3,003,092 | | 3,893,356 | | 3.08 | | 0.62 |  |
| Brazil | 311,787,435 | 14,047,197 | | 53,430,970 | | 5.84 | | 1.17 |  |
| Colombia | 74,819,933 | 9,479,085 | | 23,633,961 | | 3.17 | | 0.63 |  |
| Costa Rica | 28,495,000 | 90,063 | | 908,831 | | 31.35 | | 6.27 |  |
| Dominican Republic | 3,061,635 | 1,355,900 | | 7,734,051 | | 0.40 | | 0.08 |  |
| Ecuador | 22,584,548 | 3,216,725 | | 5,439,428 | | 4.15 | | 0.83 |  |
| El Salvador | 9,865,440 | 0 | | 3,171,089 | | 3.11 | | 0.62 |  |
| Guatemala | 27,702,599 | 4,744,037 | | 5,511,812 | | 5.03 | | 1.00 |  |
| Guyana | 6,845,397 | 612,471 | | 761,314 | | 8.99 | | 1.80 |  |
| Haiti | 12,694,515 | 5,663,054 | | 6,096,534 | | 2.08 | | 0.42 |  |
| Honduras | 10,390,087 | 2,453,780 | | 3,929,061 | | 2.64 | | 0.53 |  |
| Mexico | 114,995,375 | 2,316,147 | | 11,765,114 | | 9.77 | | 1.95 |  |
| Nicaragua | 27,861,715 | 2,908,171 | | 4,722,125 | | 5.90 | | 1.18 |  |
| Panama | 11,817,743 | 570,212 | | 1,282,716 | | 9.21 | | 1.84 |  |
| Paraguay | 17,740,426 | 891,217 | | 1,024,000 | | 17.32 | | 3.46 |  |
| Peru | 29,822,442 | 4,030,658 | | 8,336,865 | | 3.58 | | 0.72 |  |
| Suriname | 7,332,757 | 29,857 | | 29,857 | | 245.60 | | 49.12 |  |
| Venezuela | 30,614,857 | 4,427,624 | | 22,866,188 | | 1.34 | | 0.27 |  |
| **CSE Asia region** |  |  | |  | |  | |  |  |
| Afghanistan | 39,785,424 | 4,739,609 | | 24,298,341 | | 1.64 | | 0.33 |  |
| Azerbaijan | 7,438,827 | 23,987 | | 4,187,017 | | 1.78 | | 0.36 |  |
| Bangladesh | 31,962,159 | 35,688,939 | | 35,688,939 | | 0.90 | | 0.18 |  |
| Bhutan | 6,748,604 | 369,282 | | 476,065 | | 14.18 | | 2.84 |  |
| Cambodia | 76,010,917 | 10,529,860 | | 13,122,542 | | 5.79 | | 1.16 |  |
| China | 103,655,337 | 31,927,052 | | 462,068,912 | | 0.22 | | 0.04 |  |
| Georgia | 3,086,029 | 186,455 | | 477,247 | | 6.47 | | 1.29 |  |
| India | 315,345,761 | 682,186,236 | | 1,150,361,292 | | 0.27 | | 0.05 |  |
| Indonesia | 118,801,964 | 43,232,229 | | 136,135,939 | | 0.87 | | 0.17 |  |
| Iran | 36,009,612 | 81,236 | | 2,889,632 | | 12.46 | | 2.49 |  |
| Iraq | 2,587,474 | 0 | | 2,443,842 | | 1.06 | | 0.21 |  |
| Korea, Democratic People's Republic of | 13,933,014 | 21,071,016 | | 22,079,238 | | 0.63 | | 0.13 |  |
| Korea, Republic of | 3,419,891 | 1,985,023 | | 3,021,677 | | 1.13 | | 0.23 |  |
| Kyrgyzstan | 4,443,919 | 0 | | 1,910,253 | | 2.33 | | 0.47 |  |
| Lao People's Democratic Republic | 29,649,660 | 3,994,237 | | 5,585,668 | | 5.31 | | 1.06 |  |
| Malaysia | 74,791,841 | 6,437,370 | | 27,902,287 | | 2.68 | | 0.54 |  |
| Myanmar | 21,422,848 | 41,399,532 | | 45,259,934 | | 0.47 | | 0.09 |  |
| Nepal | 26,214,211 | 3,680,026 | | 24,181,770 | | 1.08 | | 0.22 |  |
| Pakistan | 52,646,287 | 45,055,815 | | 170,423,467 | | 0.31 | | 0.06 |  |
| Papua New Guinea | 68,356,093 | 4,806,992 | | 5,641,432 | | 12.12 | | 2.42 |  |
| Philippines | 71,458,429 | 36,093,217 | | 50,339,257 | | 1.42 | | 0.28 |  |
| Solomon Islands | 31,731,213 | 535,345 | | 535,345 | | 59.27 | | 11.85 |  |
| Sri Lanka | 76,629,084 | 3,514,848 | | 11,961,713 | | 6.41 | | 1.28 |  |
| Tajikistan | 10,827,394 | 0 | | 940,418 | | 11.51 | | 2.30 |  |
| Thailand | 77,309,126 | 19,852,684 | | 41,965,672 | | 1.84 | | 0.37 |  |
| Timor-Leste | 12,027,781 | 1,148,249 | | 1,148,249 | | 10.47 | | 2.09 |  |
| Turkey | 195,867,260 | 236,319 | | 1,407,057 | | 139.20 | | 27.84 |  |
| Uzbekistan | 8,633,141 | 0 | | 309,709 | | 27.88 | | 5.58 |  |
| Vanuatu | 14,822,688 | 241,027 | | 242,072 | | 61.23 | | 12.25 |  |
| Viet Nam | 42,999,511 | 12,322,264 | | 59,229,323 | | 0.73 | | 0.15 |  |
| **Additional file 2**National Funding and Populations-at-Risk (2006-2010) | | | | | | | | | |
|  | **Total Funds** | | **Stable *PfPv*PAR^[1,2]^** | | **Total *PfPv*PAR** | | **Total Per-Capita** | **Annualised Total Per-Capita** |  |
| **Africa + region** |  | |  | |  | |  |  |  |
| Angola | 227,097,704 | | 18,721,552 | | 18,982,091 | | 11.96 | 2.39 |  |
| Benin | 154,123,174 | | 9,217,872 | | 9,219,134 | | 16.72 | 3.34 |  |
| Botswana | 3,604,967 | | 1,006,958 | | 1,008,973 | | 3.57 | 0.71 |  |
| Burkina Faso | 85,913,432 | | 16,156,445 | | 16,250,123 | | 5.29 | 1.06 |  |
| Burundi | 56,101,383 | | 6,894,478 | | 6,906,225 | | 8.12 | 1.62 |  |
| Cameroon | 33,370,242 | | 19,739,212 | | 19,755,559 | | 1.69 | 0.34 |  |
| Cape Verde | 1,642,214 | | 0 | | 199,231 | | 8.24 | 1.65 |  |
| Central African Republic | 12,973,662 | | 4,504,553 | | 4,506,361 | | 2.88 | 0.58 |  |
| Chad | 32,944,895 | | 10,619,917 | | 11,508,739 | | 2.86 | 0.57 |  |
| Comoros | 6,051,684 | | 665,612 | | 665,793 | | 9.09 | 1.82 |  |
| Congo | 21,844,302 | | 3,760,028 | | 3,760,028 | | 5.81 | 1.16 |  |
| Cote d'Ivoire | 123,999,987 | | 21,570,550 | | 21,571,294 | | 5.75 | 1.15 |  |
| Democratic Republic of Congo | 244,690,548 | | 64,039,086 | | 64,071,448 | | 3.82 | 0.76 |  |
| Djibouti | 4,274,645 | | 25,131 | | 390,801 | | 10.94 | 2.19 |  |
| Equatorial Guinea | 29,289,299 | | 669,430 | | 669,430 | | 43.75 | 8.75 |  |
| Eritrea | 34,440,847 | | 3,271,074 | | 4,412,793 | | 7.80 | 1.56 |  |
| Ethiopia | 450,113,026 | | 64,370,653 | | 66,883,826 | | 6.73 | 1.35 |  |
| Gabon | 19,643,872 | | 1,501,014 | | 1,501,014 | | 13.09 | 2.62 |  |
| Ghana | 265,651,464 | | 24,235,510 | | 24,338,799 | | 10.91 | 2.18 |  |
| Guinea | 27,607,416 | | 10,248,058 | | 10,323,455 | | 2.67 | 0.53 |  |
| Guinea-Bissau | 12,034,475 | | 1,645,005 | | 1,647,008 | | 7.31 | 1.46 |  |
| Kenya | 419,505,587 | | 31,499,901 | | 31,943,492 | | 13.13 | 2.63 |  |
| Liberia | 88,373,372 | | 4,102,036 | | 4,102,050 | | 21.54 | 4.31 |  |
| Madagascar | 230,323,758 | | 19,001,651 | | 19,001,651 | | 12.12 | 2.42 |  |
| Malawi | 306,828,965 | | 15,688,159 | | 15,688,159 | | 19.56 | 3.91 |  |
| Mali | 120,180,325 | | 12,453,553 | | 13,361,648 | | 8.99 | 1.80 |  |
| Mauritania | 13,396,325 | | 1,934,061 | | 3,098,732 | | 4.32 | 0.86 |  |
| Mozambique | 272,567,541 | | 23,402,580 | | 23,404,038 | | 11.65 | 2.33 |  |
| Namibia | 59,910,225 | | 1,366,966 | | 1,478,276 | | 40.53 | 8.11 |  |
| Niger | 59,880,413 | | 14,274,886 | | 15,884,893 | | 3.77 | 0.75 |  |
| Nigeria | 614,086,653 | | 157,623,504 | | 158,254,621 | | 3.88 | 0.78 |  |
| Rwanda | 244,973,007 | | 6,569,512 | | 6,589,807 | | 37.17 | 7.43 |  |
| Sao Tome and Principe | 5,713,648 | | 163,315 | | 163,315 | | 34.99 | 7.00 |  |
| Saudi Arabia | 139,699,086 | | 338,184 | | 1,580,302 | | 88.40 | 17.68 |  |
| Senegal | 174,345,587 | | 12,641,313 | | 12,865,669 | | 13.55 | 2.71 |  |
| Sierra Leone | 27,717,062 | | 5,836,369 | | 5,836,573 | | 4.75 | 0.95 |  |
| Somalia | 23,864,657 | | 8,591,307 | | 9,357,579 | | 2.55 | 0.51 |  |
| South Africa | 261,752,409 | | 5,525,261 | | 16,631,122 | | 15.74 | 3.15 |  |
| Sudan | 195,157,935 | | 35,365,559 | | 43,202,010 | | 4.52 | 0.90 |  |
| Swaziland | 10,921,255 | | 0 | | 348,180 | | 31.37 | 6.27 |  |
| Tanzania | 591,534,332 | | 43,587,912 | | 43,606,820 | | 13.57 | 2.71 |  |
| The Gambia | 33,173,680 | | 1,746,987 | | 1,751,006 | | 18.95 | 3.79 |  |
| Togo | 117,845,431 | | 6,771,715 | | 6,773,815 | | 17.40 | 3.48 |  |
| Uganda | 307,434,809 | | 32,274,215 | | 32,313,772 | | 9.51 | 1.90 |  |
| Yemen | 30,656,020 | | 8,689,430 | | 21,925,986 | | 1.40 | 0.28 |  |
| Zambia | 189,682,667 | | 13,253,422 | | 13,253,422 | | 14.31 | 2.86 |  |
| Zimbabwe | 74,083,032 | | 12,469,653 | | 12,472,248 | | 5.94 | 1.19 |  |
|  |  |  | |  | |  | |  |  |
| All currency quoted as US$ ^[1]^*PfPv* = Combined *P.falciparum* and *P.vivax* risk ^[2]^PAR = Population at risk | | | | | | | | | |
