# Supplementary material for: Funding for malaria control 2006–2010: A comprehensive global assessment
Source: Malar J. 2012 Jul 28;11:246. doi: 10.1186/1475-2875-11-246 (PMC3444429; doi:10.1186/1475-2875-11-246)
Supplement: Additional file 3 — Multi-Country Grants and Their Divisions. [file 1475-2875-11-246-S3.doc]

| **Additional file 3**Multi-Country Grants and Their Divisions | | | | | | | |
| --- | --- | --- | --- | --- | --- | --- | --- |
| **Grant** | **Countries** | | | | **Ratio of Funding** | | **Notes** |
| Multicountry West Pacific [Global Fund^[1]^] | Solomon Islands, Vanuatu | | | | 2:1 | | Three grants |
| Multicountry Africa (RMCC) [Global Fund] | Mozambique, Swaziland, South Africa | | | | 77:12:11 | | Two grants: First divided between all three countries, second to Mozambique only |
| Multicountry Americas (Andean) [Global Fund] | Colombia, Peru, Venezuela, Ecuador | | | | 46:32:12:9 | | Divided in proportion to number of municipalities where API> 10 in 50% (listed within grant proposal) |
| Senegal River Basin Multi-Purpose Water Resources Development Project [World Bank] | Mauritania, Mali, Senegal, Guinea | | | | 29:27:27:16 | | Divided as per the entire Project, of which malaria was just a subset |
|  | | | | | | | |
| ^[1]^The Global Fund to Fight AIDS, Tuberculosis and Malaria | | | | | | | |
|  | |  |  |  | |  |  |
