# Supplementary material for: Funding for malaria control 2006–2010: A comprehensive global assessment
Source: Malar J. 2012 Jul 28;11:246. doi: 10.1186/1475-2875-11-246 (PMC3444429; doi:10.1186/1475-2875-11-246)
Supplement: Additional file 4 — Funding patterns in selected DAC donors. [file 1475-2875-11-246-S4.doc]

| **Additional file 4** Funding patterns in selected DAC donors | | | |  | | | |
| --- | --- | --- | --- | --- | --- | --- | --- |
| **DAC Donor** | **Recipient** | **Amount Received** |  |  | | | |
| Portugal | Angola* | 61,965 | 100% Colonial Heritage |  | | | |
|  | Cape Verde* | 22,551 |  |  | | | |
|  | Mozambique* | 101,544 |  |  | | | |
|  | Sao Tome and Principe* | 167,673 |  |  | | | |
| Australia | Democratic Republic of Congo | 141 |  |  | | | |
|  | Uganda | 224 |  |  | | | |
|  | Indonesia^†^ | 19,174 | 99.99% South East Asia |  | | | |
|  | Solomon Islands^†^ | 9,846,432 |  |  | | | |
|  | Papua New Guinea^†^ | 204,035 |  |  | | | |
|  | Philippines^†^ | 2,332,532 |  |  | | | |
|  | Vanuatu^†^ | 3,877,665 |  |  | | | |
| United Kingdom | Burundi | 4,156,121 |  |  | | | |
|  | Ethiopia | 1,059,525 |  |  | | | |
|  | Ghana* | 20,931,931 | 96.95% Commonwealth |  | | | |
|  | Kenya* | 68,589,063 |  |  | | | |
|  | Mozambique* | 11,908,952 |  |  | | | |
|  | Nigeria* | 30,404,187 |  |  | | | |
|  | Sierra Leone* | 9,344,260 |  |  | | | |
|  | Somalia* | 2,053,785 |  |  | | | |
|  | Sudan* | 3,948,309 |  |  | | | |
|  | Tanzania* | 6,904,681 |  |  | | | |
|  | Uganda* | 1,707,050 |  |  | | | |
|  | Zambia* | 8,602,317 |  |  | | | |
|  | Zimbabwe* | 1,404,367 |  |  | | | |
| USA | Brazil | 144,895 |  |  | | | |
|  | Burkina Faso | 4,983,067 |  |  | | | |
|  | Burundi | 5,724,882 |  |  | | | |
|  | Cameroon | 50,000 |  |  | | | |
|  | Congo | 100,000 |  |  | | | |
|  | Guatemala | 174,858 |  |  | | | |
|  | Peru | 4,297,981 |  |  | | | |
|  | Sudan | 8,788,756 |  |  | | | |
|  | Zimbabwe | 397,165 |  |  | | | |
|  | Angola^‡^ | 57,488,818 | 90.13% PMI Supported |  | | | |
|  | Benin^‡^ | 25,155,814 |  |  | | | |
|  | Democratic Republic of Congo^‡^ | 24,146,946 |  |  | | | |
|  | Ethiopia^‡^ | 43,542,000 |  |  | | | |
| **DAC Donor** | **Recipient** | **Amount Received** |  |  | | | |
| USA (continued) | Ghana^‡^ | 44,829,607 |  |  | | | |
|  | Kenya^‡^ | 44,878,363 |  |  | | | |
|  | Liberia^‡^ | 17,304,015 |  |  | | | |
|  | Madagascar^‡^ | 51,306,643 |  |  | | | |
|  | Malawi^‡^ | 52,288,143 |  |  | | | |
|  | Mali^‡^ | 31,047,089 |  |  | | | |
|  | Mozambique^‡^ | 54,836,225 |  |  | | | |
|  | Nigeria^‡^ | 21,308,338 |  |  | | | |
|  | Rwanda^‡^ | 49,308,423 |  |  | | | |
|  | Senegal^‡^ | 47,663,811 |  |  | | | |
|  | Tanzania^‡^ | 97,761,674 |  |  | | | |
|  | Uganda^‡^ | 57,149,668 |  |  | | | |
|  | Zambia^‡^ | 34,733,802 |  |  | | | |
| Belgium | Benin | 106,739 |  |  | | | |
|  | Uganda | 557,930 |  |  | | | |
|  | Burundi* | 610,856 | 87.5% Colonial Heritage |  | | | |
|  | Democratic Republic of Congo* | 296,932 |  |  | | | |
|  | Rwanda* | 3,743,271 |  |  | | | |
| France | Bolivia | 1,433 |  |  | | | |
|  | Mozambique | 128,116 |  |  | | | |
|  | Peru | 735,440 |  |  | | | |
|  | Benin* | 2,081,782 | 81.64% Colonial Heritage |  | | | |
|  | Burkina Faso* | 673,711 |  |  | | | |
|  | Cameroon* | 231,699 |  |  | | | |
|  | Cote d’Ivoire* | 1,941 |  |  | | | |
|  | Laos* | 9,618 |  |  | | | |
|  | Mali* | 2,351 |  |  | | | |
|  | Senegal* | 844,933 |  |  | | | |
| The dotted line distinguishes those recipients that have a link to the donor nation, with reasoning stated in the rightmost column. The percentage value stated represents the proportion of the total amount disbursed by these donor nations received by nations with links. All currency quoted as US$ *represents former colony/commonwealth nation ^†^Pacific and South East Asia ^‡^PMI recipient | | | |  | | | |
|  |  |  | | |  |  |  |
